# Evaluation of the analytical performance of six rapid diagnostic tests for the detection of HIV-1 and 2 in Lubumbashi, Democratic Republic of Congo

**DOI:** 10.11604/pamj.2025.52.22.44084

**Published:** 2025-09-17

**Authors:** Bernard Kalunga-Tompa, Arsène Kabamba-Tshikongo, Rachel Mujinga-Kayembe, Benoit Kabamba-Mukadi, Jean-Marie Liesse-Iyamba, Albert Longanga-Otshudi

**Affiliations:** 1 Laboratoire de Biologie Clinique, Faculté des Sciences Pharmaceutiques, Université de Lubumbashi, Commune de Kampemba, Lubumbashi, Democratic Republic of Congo,; 2 Centre d´Excellence et d´Expertise des hépatites virales et autres pathologies, Lubumbashi, Democratic Republic of Congo,; 3 Institut de Recherche Expérimentale et Clinique, Pôle de Microbiologie, Université Catholique de Louvain, Bruxelles, Belgique,; 4 Laboratoire de Microbiologie, Faculté des Sciences Pharmaceutiques, Université de Kinshasa, Kinshasa, Democratic Republic of Congo

**Keywords:** Screening, diagnosis, analytical performance, rapid diagnostic tests

## Abstract

Human immunodeficiency virus (HIV) infection constitutes a major public health concern worldwide. According to UNAIDS estimates, Africa would be the most affected continent in the world, with around 25.7 million cases recorded, and the Democratic Republic of Congo (DRC) would be among the 22 countries in the world with the heavy burden of HIV. Screening constitutes an important lever in the prevention of this infection. In developing countries such as the DRC, rapid diagnostic tests (RDTs) are widely used in screening for HIV infection. Still, these RDTs might have a serious problem of analytical performance, which could compromise the prevention and medical management of HIV infection. To date, no study has been carried out in Lubumbashi to assess the quality of these RDTs. This study aimed to evaluate the analytical performance of RDTs used in Lubumbashi for the screening and diagnosis of HIV infection. A total of 200 serum samples (100 HIV positive samples and 100 HIV negative samples) were tested simultaneously by six locally used brands of RDTs and by the Liaison XL Murex assay used as a reference test. All six evaluated RDTs showed analytical performance in line with WHO standards, with a sensitivity and NPV of 100%, a specificity and PPV of 99% and a Kappa coefficient of 99%. The Unigold RDT mark, having presented a high detection limit, could be proposed as the RDT of first choice in HIV screening in Lubumbashi.

## Introduction

Human immunodeficiency virus (HIV) is a retrovirus [1]. It is the causative agent of AIDS. HIV/AIDS constitutes a major public health problem worldwide. In 2021, according to UNAIDS estimates, there would be around 39 million people living with HIV in the world, including 1.7 million new cases of infection. Around 25.4 million people had access to antiretroviral therapy, and 690,000 people died from AIDS-related illnesses. The total number of Africans living with HIV/AIDS was 25.7 million in 2021 [2].

The Democratic Republic of Congo (DRC) is among 22 countries in the world with a heavy burden of HIV/AIDS. According to estimates from the sentinel sero-surveillance system (2013) as well as the demographic and health survey (2013/2014), the HIV epidemic is of the generalized type; the DRC has a prevalence of 1.2% in the general population. Estimates from the national AIDS control program (PNLS) indicate that between 2013 - 2014, nearly 450,000 people were living with HIV [3]. The Joint United Nations Programme on HIV/AIDS (UNAIDS) has set itself the objectives to be achieved by 2030. These objectives, called 95-95-95, stipulate that by 2030: 95% of infected people must know their immunological status via mass screening, 95% of HIV-positive people must have access to antiretroviral treatment, and 95% of people on antiretroviral treatment (ARV) must have their viral load permanently suppressed [2]. Given the magnitude of this infection, its correct detection and diagnosis are a public health priority [4].

The last decade has seen the emergence of numerous technologies that allow the diagnosis of infectious diseases to be carried out quickly and reliably [5]. Rapid diagnostic tests (RDTs), whether of the “immuno-chromatographic” or “immuno-filtration” type, highlighting specific antigens or antibodies, have given a boost to the fight against tropical infections [4]. It should also be noted that there are few adequate quality control systems accessible to countries with limited resources. Rapid diagnostic tests for the detection of anti-HIV antibodies (RDTs) are widely used in developing countries (DCs) or in countries with low social coverage [6]. The use of these tests is simple, however, although the manufacturers of these tests report very appreciable levels of reliability, their analytical performance is often not determined independently in developing countries, such as is the case for the DRC. Assurance on the quality of RDTs is, in part, the first step for the accurate diagnosis of HIV infections. Rapid diagnostic tests with poor analytical performance result in many cases of false negative or false positive results. Therefore, this study aimed to determine the analytical performance of RDTs used in HIV screening and diagnosis in Lubumbashi.

## Methods

**Type, period, and site of study:** this was a cross-sectional descriptive study carried out over the period from May 25, 2020, to February 25, 2021. The evaluation of the analytical performance of RDTs used in HIV screening in Lubumbashi took place at the Clinical Biology laboratory of the Faculty of Pharmaceutical Sciences, University of Lubumbashi, and at the laboratory of the Institute of Experimental and Clinical Research (IREC), Microbiology center of the Catholic University of Louvain (UCLouvain).

**Composition of the reference sample panel:** the study involved a total of 200 blood sera, of which 100 were positive and 100 were negative for HIV. Positive serum samples were taken from patients infected with HIV and monitored at the care center for people living with HIV at Sendwe Hospital. Negative serum samples were taken from blood donors. Serum samples were selected using the combination of three RDTs included in the national algorithm (Alere Determine™ HIV-1 and 2, Vikia® HIV-1 and 2, and Unigold™ HIV). Subsequently, they were frozen and stored at -20°C.

**Rapid diagnostic test (RDT):** six marks of RDTs were evaluated in this study. They were: Alere Determine™ HIV-1 and 2 (USA), Vikia® HIV-1 and 2 (France), Unigold™ HIV (Ireland), SD Bioline HIV-1 and 2 3.0 (Korea), Haelgen® (Japan), First Response HIV-1-2-0 (India). Out of the six evaluated RDTs, three marks have been included in the national algorithm (Alere™ Determine™ HIV-1 and 2, Vikia® HIV-1 and 2, Unigold™ HIV). The three others are marketed in Lubumbashi. The RDTs are qualitative tests based on the lateral flow immunochromatographic technique for the detection of HIV-1 and 2 antibodies in serum samples.

**The Liaison XL Murex HIV Ab/Ag (Diasorin, Saluggia, Italy, REF310260):** the Liaison XL is a direct, sandwich, two-stage incubation test chemiluminescent immunoassay (CLIA) for the qualitative determination of antibodies and specific antigens directed against human immunodeficiency virus type 1 (group M and group O) and/or human immunodeficiency virus type 2 in human serum or plasma samples [7]. The analytical performance evaluation of each RDT was carried out by systematically and in parallel testing 100 serum samples from the HIV-positive panel and 100 serum samples from the HIV-negative panel, using the six RDTs and Liaison XL as the reference method. Therefore, 1400 tests were carried out to evaluate the analytical performance of these six RDTs. All tests were performed according to the manufacturer´s recommendations. Characteristics of the evaluated RDTs and the reference test are listed in Table 1. The following analytical performance indicator parameters were evaluated for each RDT with a 95% confidence interval: sensitivity, specificity, positive predictive value (PPV), negative predictive value (NPV), and the Kappa concordance coefficient. A result was considered a true positive (TP) in the event of reactivity on both RDT and Liaison XL, and as a true negative (TN) in the event of no reactivity on both tests. On the other hand, a result was considered a false positive (FP) in the event of no reactivity on Liaison XL and the presence of reactivity on RDT. In the same way, a result was considered as a false negative (FN) in the event of the absence of reactivity on RDT and reactivity on Liaison XL.

**Table 1:** characteristics of RDTs evaluated according to manufacturers

RDT	Sample	Composition	Technique	Discriminant	ST (C)	TD (min)	SV (μL)	Test price ($)	Firms	Firm’s countries
A	Se, Pl, TB	Automatic analyzer	Chemiluminescence			60	300	3.00	Diasorin S.p.A	Italy
B	Se, Pl, TB	Gp 41, gp 36, p 24	Immuno-chromatography	Presence	4-30		20	1.41	Premier Medical Corporation Limited	India
C	Se, Pl, TB	Gp 41, gp 36, p 24	Immuno-chromatography	Absence	2-30	10-20	20	1.00	SD Standard Diagnostics, inc	Korea
D	Se, Pl, TB	Gp 41, gp 36,	Immuno-chromatography	Absence	4-30	30	75	0.78	Biomerieux	France
E	Se, Pl, TB	Gp 41, gp 36, gp 120	Immuno-chromatography	Absence	2-27	10-12	60	1.00	Trinity Biotech Plc	Ireland
F	Se, Pl, TB	Gp 41, gp 36, gp 120	Immuno-chromatography	Absence	2-30	15-30	50	0.75	Healgen Scientific Limited Liability Company	USA
G	Se, Pl, TB	Gp 41, gp 36, gp 120	Immuno-chromatography	Absence	2-30	15-60	50	0.75	Alere Medical Co., Ltd	Japan

A: Liaison XL® MUREX HIV Ab/Ag; B: first Response HIV 1-2-0; C: SD Bioline HIV 1/2 3.0; D: Vikia HIH 1 and 2; E: unigoldTM HIV; F: Healgen; G: alere determine HIV-1 and 2; ST: storage temperature; TD: test duration (minutes); SV: sample volume; Se: serum; Pl: plasma; TB: total blood; gp: glycoprotein; p: protein; RDT: rapid diagnostic tests

**Limit of detection (LoD) study:** the detection limit of RDTs compared to the reference method was evaluated by preparing a 10-fold dilution series of three anti-HIV positive serum samples, 6 dilutions for each sample. The first serum sample had a viral load of 27791 C/mL, the second serum sample had a viral load of 50 C/mL, and the third sample had an undetectable viral load. Each dilution was tested for the detection of anti-HIV antibody simultaneously by RDTs and by Liaison XL MUREX HIV Ab/Ag.

**Statistical analysis:** the collected data in relation to this study were entered into the Epi-data software and exported into the Epi-Info software version 7.2.5.0 for statistical analysis (Student's t-test). Sensitivity, specificity, positive predictive value (PPV), and negative predictive value (NPV) for each RDT were assessed, and their respective 95% confidence intervals (95%CI) were calculated. The p-value was calculated to determine the existence of a statistically significant link between the study variables. Statistical significance: p < 0.05. The Kappa coefficient was calculated to assess the degree of agreement of the evaluated RDTs with the reference test.

**Ethical committee approval:** free and informed consent was previously obtained from the participants in this study. This work obtained the approval from the Medical Ethics Committee of the University of Lubumbashi, under approval No: UNILU/CEM/125/2022.

## Results

**Determination of the analytical performance of rapid diagnostic tests:** the evaluation of the six rapid diagnostic tests for HIV infection showed that all the RDTs evaluated could be used with whole blood, plasma, or even serum. The RDTs studied had a sensitivity and negative predictive value (NPV) of 100%, specificity and positive predictive value (PPV) of 99%. The Kappa coefficient value was > 99.2% (Table 2).

**Table 2:** sensitivity, specificity, PPV, and NPV of 6 analyzed tests

RDTs	Total (N)	TP; a	FP; b	TN; c	FN; d	% sensitivity (CI 95%)	% specificity (CI 95%)	% PPV (CI 95%)	% NPV (CI 95%)	% Kappa (CI 95%)
A	200	99	1	100	0	100.0 (98.9 – 100.0)	99.0 (98.6 – 100.0)	99.0 (98.8 – 100.0)	100.0 (99.1 – 100.0)	99.2 (98.9 – 100.0)
B	200	99	1	100	0	100.0 (98.9 – 100.0)	99.0 (98.6 – 100.0)	99.0 (98.8 – 100.0)	100,0 (99.1 – 100.0)	99.2 (98.9 – 100.0)
C	200	99	1	100	0	100.0 (98.9 – 100.0)	99.0 (98.6 – 100.0)	99.0 (98.8 – 100.0)	100.0 (99.1 - 100,0)	99.2 (98.9 – 100.0)
D	200	99	1	100	0	100.0 (98.9 – 100.0)	99.0 (98.6 – 100.0)	99.0 (98.8 – 100.0)	100.0 (99.1 – 100.0)	99.2 (98.9 – 100.0)
E	200	99	1	100	0	100.0 (98.9 – 100.0)	99.0 (98.6 – 100.0)	99.0 (98.8 – 100.0)	100,0 (99.1 – 100.0)	99.2 (98.9 – 100.0)
F	200	99	1	100	0	100.0 (98.9 – 100.0)	99.0 (98.6 – 100.0)	99.0 (98.8 – 100.0)	100.0 (99.1 – 100.0)	99.2 (98.9 – 100.0)
G	200	99	1	100	0	100.0 (98.9 – 100.0)	99.0 (98.6 – 100.0)	99.0 (98.8 – 100.0)	100.0 (99.1 – 100.0)	99.2 (98.9 – 100.0)

**A**: Liaison XL® MUREX HIV Ab/Ag); **B**: first response VIH 1-2-0; **C**: SD bioline HIV 1 and 2 3.0; **D**: Vikia HIH 1 and 2; **E**: unigoldTM HIV; **F**: Healgen; **G**: Alere determine HIV-1 and 2**; TP**: true positive, **FP**: false positive, **TN**: true negative, **FN**: false negative, **CI**: confidence interval, **PPV**: predictive positive value, NPV: negative predictive value; CI : confidence interval ; RDT: rapid diagnostic tests

**Study of the limit of detection (LoD):** in this study, the Unigold test was sensitive for the detection of anti-HIV antibodies up to the fourth dilution for the first sample with a viral load of 27791 C/mL and the second sample with a viral load of 50 C/mL. Regarding the third sample with an undetectable viral load, the Unigold test was sensitive up to the third dilution. The Healgen test was sensitive up to the second dilution for the first and second samples, while for the third sample, the Healgen test was sensitive only to the first dilution. The Alere Determine^TM^ test was only sensitive at the first dilution for all three evaluated samples. The Vikia test was sensitive to the first dilution for the first and second samples. The first response test and SD Bioline showed no sensitivity for the three analyzed samples (Table 3).

**Table 3:** limit of detection (LoD) study

Viral load = 27791 C/mL						Viral load = 50 C/mL					Undetectable viral load				
RDT	101	102	103	104	105	101	102	103	104	105	101	102	103	104	105
A	+	+	+	+	-	+	+	+	+	-	+	+	+	-	-
B	-	-	-	-	-	-	-	-	-	-	-	-	-	-	-
C	-	-	-	-	-	-	-	-	-	-	-	-	-	-	-
D	+	-	-	-	-	+	-	-	-	-	-	-	-	-	-
E	+	+	+	+	-	+	+	+	+	-	+	+	+	-	-
F	+	+	-	-	-	+	+	-	-	-	+	-	-	-	-
G	+	-	-	-	-	+	-	-	-	-	+	-	-	-	-

A Liaison XL® MUREX HIV Ab/Ag); B: first response VIH 1-2-0; C: SD Bioline HIV 1 and 2 3.0; D: Vikia HIH 1 and 2; E : unigoldTM HIV; F: healgen; G: alere determine HIV-1 and 2, +: reactive test, -: non-reactive test ; RDT: rapid diagnostic tests

## Discussion

**Characteristics of the tests evaluated according to the manufacturers:** rapid diagnostic tests have several advantages, including ease of use, possibility of storage at room temperature, and ease of access. They have good sensitivity and specificity during the chronic phase of infection [8]. In countries with limited resources, these tests have revolutionized HIV screening and require neither highly qualified personnel nor very heavy equipment [9]. On the other hand, RDTs have some disadvantages, such as the lack of sensitivity in the early phase of infection (i.e. the serological window phase), and sometimes subjective reading of the results [8]. Currently, some studies show the limitations of rapid diagnostic tests in the diagnosis of HIV; a poor procedure can lead to extremely low levels of sensitivity [10]. The RDT analysis time is reduced (15-30 min instead of 60 min for the Liaison XL® MUREX HIV Ab/Ag), and the sample volume required for analysis is reduced (20 µL - 75 µL instead of 350 µL [5]. The cost of the analysis is ten times cheaper than analyses on Liaison XL [11]. In addition, the possibility of using whole blood is an important advantage for screening peripheral centers (no possibility of centrifugation or decantation of serum), as suggested by the guidelines for the evaluation of HIV screening techniques in Africa [4]. These different characteristics make RDTs important tools for the diagnosis of HIV in areas where there is no infrastructure and specialized laboratory technicians [11]. However, despite the high costs of its analyses, the XL® MUREX HIV Ab/Ag Liaison method remains more reliable than RDTs.

**The performance of rapid diagnostic tests:** at the end of this study, which focused on the evaluation of the analytical performance of the six RDTs, it turned out that all the evaluated RDTs presented a sensitivity of 100% and a specificity of 99%. Different studies carried out around the world to evaluate the analytical performance of RDTs in the detection of HIV have presented different levels of performance. Some performance levels are either higher or lower than those obtained in this study, and other performance levels corroborate those obtained in this study. Concerning the Alere Determine™ HIV-1 and 2 RDTs, Dagnra *et al*. [12] obtained a sensitivity of 88.9% and a specificity of 97.0% in a study carried out at the National Reference Center for HIV/AIDS/STI tests in Lomé (Togo). On the other hand, Djimadoum *et al*. [13] found a sensitivity of 100% and a specificity of 100% in their study.

Regarding first response RDTs, Dagnra *et al*. [12] found a sensitivity of 99% and a specificity of 98.1% in a study carried out among patients hospitalized at the Tokoin University Hospital in Lomé. Regarding Bioline RDTs, Djimadoum *et al*. [13], found in their study a sensitivity of 99% and a specificity of 100%. Furthermore, in a study carried out in 5 African countries to evaluate the performance of Unigold RDTs in HIV screening; a sensitivity of 96.2% and specificity of 100% for Arua (Uganda); a sensitivity of 99.6% and specificity of 99.7% for Homa Bay (Kenya), a sensitivity of 100% and specificity of 89.2% for Baraka (DRC). In the same study, the results of the Vikia RDT presented the following performances: a sensitivity of 100% and specificity of 99.3% for Conakry (Guinea); a sensitivity of 100% and specificity of 98.1% for Arua (Uganda), a sensitivity of 100% and specificity of 97.2% for Baraka (DRC). This difference in results on the analytical performance of RDTs shows that they do not guarantee absolute sensitivity and specificity. This also demonstrates the importance of quality controls locally. According to some authors [14-16], the differences in sensitivity and specificity encountered in different marks of RDTs could be attributed to variations in the capacity of the RDT to detect early seroconversion due to genetic polymorphism of HIV or to the use of different antigens/epitopes or even to the study sample size. On the other hand, it has been advised to validate RDTs based on the geographic distribution of their use to ensure that the test is adequately sensitive to locally circulating HIV types [17].

Regarding the detection limit, although all the evaluated RDTs presented a sensitivity of 100%, it appears that only the Unigold marks of RDTs presented a sensitivity equivalent to that of the reference method (Liaison XL). It should be noted that RDTs used in HIV testing have lower sensitivity than some conventional tests, particularly during the early phase of infection [18]. False negative results were also observed in patients with advanced disease and in patients on antiretroviral treatment. As part of this study, the six RDTs evaluated met the WHO reliability criteria, namely a sensitivity > 99% and a specificity > 95% [19].

**Limitations of the study:** the reference test used in this study is the MUREX HIV Ab/Ag XL® Liaison test. It detects both antigens and antibodies of HIV-1 and 2. It should be interesting in the future to use a genomic amplification test, which would allow the detection of viral RNA in the case of HIV. Moreover, as part of this study, only six brands of RDTs were evaluated. It would be necessary to work on a larger number of RDTs available on the Lubumbashi market.

## Conclusion

The objective of this study was to evaluate the analytical performance of six RDTs commonly used in Lubumbashi for the screening and diagnosis of HIV-1 and 2. Based on the results obtained and considering the guidelines for evaluation of HIV testing techniques in Africa, all RDTs evaluated in this study presented analytical performances meeting the standards of the World Health Organization. Their use in HIV screening in Lubumbashi should be encouraged. The Unigold RDT could be used as a first line in the national screening algorithm and for transfusion safety because it has proven to be more efficient in terms of sensitivity compared to other tests used in Lubumbashi.

### What is known about this topic

- Several studies have been carried out to evaluate the analytical performance of rapid diagnostic tests for human immunodeficiency virus (HIV-1 and HIV-2);
- Sensitivity, specificity, PPV, and NPV are important indicator parameters to evaluate the analytical performance of HIV RDT.

### What this study adds

- Out of the six evaluated HIV RDTs, our study highlighted the best analytical quality of “Unigold RDT”; therefore, we suggested its use as first line in the HIV national screening algorithm and for transfusion safety;
- All evaluated HIV RDTs used in the various medical structures of Lubumbashi were in line with the standards set by the WHO.
